# A Case of Abdominal Closure Using Endoscopic Anterior Component Separation Following Open Abdomen Management for Colon Perforation

**DOI:** 10.70352/scrj.cr.25-0614

**Published:** 2026-03-27

**Authors:** Chika Morita, Naoya Matsumoto, Hiroto Miyanaga, Keitaro Kakinoki, Tetsuya Sakai, Hideki Sakahira

**Affiliations:** Department of General and Gastroenterological Surgery, Hyogo Prefectural Harima Himeji General Medical Center, Himeji, Hyogo, Japan

**Keywords:** open abdomen management, endoscopic anterior component separation, surgical site occurrence

## Abstract

**INTRODUCTION:**

In open abdomen management (OAM), consistent early closure is crucial to avoid complications and improve short- and long-term outcomes. Open anterior component separation (OCS) has been reported to be a method for early definitive closure after OAM.

Originally, OCS is a common treatment option for incisional hernias. However, a major drawback is the high incidence of surgical site occurrences (SSOs) owing to impaired blood flow. This is caused by damage to the perforating branches supplying the skin from the rectus abdominis muscle, necessitating extensive subcutaneous dissection. To overcome these limitations, endoscopic anterior component separation (ECS) was developed. By eliminating the need for extensive subcutaneous dissection, perforating branches were preserved in the abdominal wall, significantly reducing SSOs.

While reports of OCS being performed for definitive closure after OAM are occasionally seen, there have been no previous case reports applying ECS. We therefore present this case.

**CASE PRESENTATION:**

A man in his 50s was followed up for multiple visceral artery aneurysms. Surgery was performed for hepatic and splenic artery aneurysms. The patient developed a postoperative pancreatic fistula that progressed favorably. However, on POD 22, the patient developed colonic perforation, necessitating emergency surgery. Owing to significant intestinal edema associated with inflammation and adhesion lysis, closure was difficult, leading to OAM. A second examination was performed 2 days later, but closure remained difficult. The abdomen was closed using ECS. There were no SSOs. After treatment for an intra-abdominal abscess, the patient was discharged 40 days after initial surgery for colon perforation. Seventeen months postoperatively, the patient remained complication-free with well-maintained abdominal wall integrity.

**CONCLUSIONS:**

OCS, which enables mesh-free closure after trauma or infection surgery, is highly useful. ECS can also be applied for early definitive closure after OAM, offering potential for minimally invasive care and reduced SSO. While no previous reports exist on applying ECS after OAM, its relatively simple technique warrants wider adoption, the further accumulation of data, and an evaluation of cases.

## Abbreviations

ECS
: endoscopic anterior component separation
OAM
: open abdomen management
OCS
: open anterior component separation
SSO
: surgical site occurrence
TAR
: transversus abdominis muscle release

## INTRODUCTION

ECS has demonstrated favorable outcomes in the repair of incisional hernias. By contrast, OCS is associated with a high incidence of SSOs, including surgical site infection, wound dehiscence, seroma, hematoma, skin necrosis, and delayed wound healing. In this case, ECS was performed following OAM for colon perforation. There were no SSOs and the postoperative course was favorable. To our knowledge, there have been no previous reports of ECS being utilized after OAM.

## CASE PRESENTATION

The patient was a man in his 50s, with a history of surgery for Stanford type A aortic dissection. The patient was also followed for multiple visceral artery aneurysms. Owing to progressive enlargement of the hepatic and splenic artery aneurysms, elective surgery was indicated.

### Surgery for multiple visceral artery aneurysms

A midline abdominal incision was made. The aneurysm of the common hepatic artery was resected, and a bypass was constructed between the splenic and proper hepatic arteries. The splenic artery aneurysm was resected and splenectomy was performed. Dense adhesions were observed around the aneurysms, making the dissection challenging. Pancreatic injury occurred during the procedure and was repaired intraoperatively. Although a postoperative pancreatic fistula developed, it was successfully managed using drainage. However, on POD 22, the patient developed abdominal pain and was diagnosed with a colon perforation, necessitating emergency surgery.

### Emergency surgery for colon perforation

Perforation was identified in the transverse colon near the splenic flexure. Mobilization was difficult because of inflammation caused by pancreatic leakage. Consequently, extended right hemicolectomy and partial small bowel resection were performed because of intraoperative injury. OAM was performed because primary fascial closure was not possible due to marked tissue edema, using −75 mmHg negative pressure therapy with 3M AbThera Advance Open Abdomen Dressing Kit (3M company, St. Paul, Minnesota, USA). The operative details were as follows: operative time 10 h 11 min; blood loss (including ascites) 2623 mL; urine output 970 mL; infusion volume 10800 mL; and transfusion of 8 units of red blood cells and 10 units of fresh frozen plasma.

### Second-look surgery

**Fig. 1:**
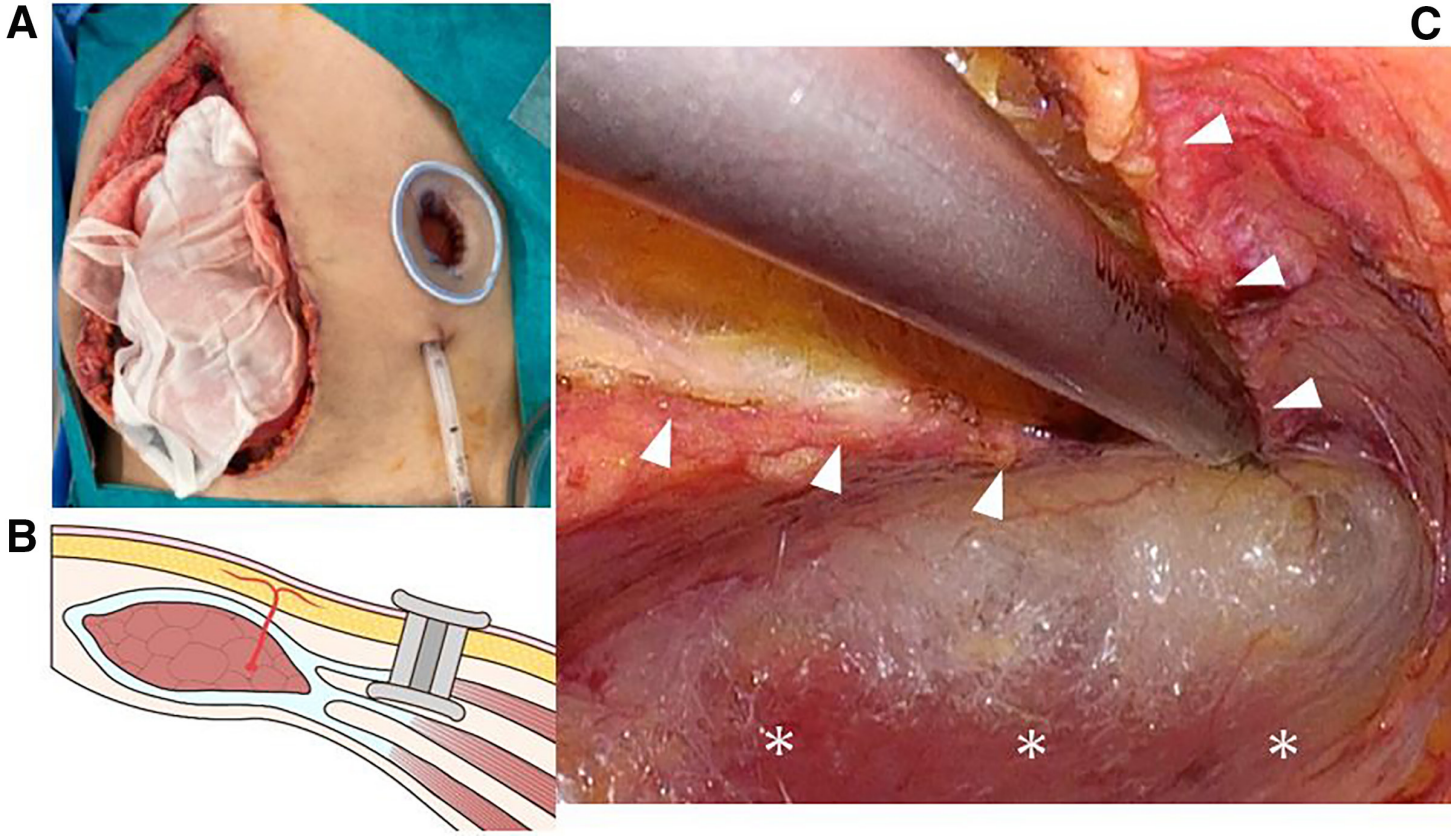
Surgical procedure (left side). (**A**) Subcutaneous dissection was performed under direct vision up to the surface of the internal oblique muscle, and a Lap-Protector was inserted into this space. (**B**) A Lap-Protector was inserted between the external and internal oblique muscles. (**C**) Under endoscopic guidance, an external oblique aponeurosis was incised from the subcostal region to the inguinal ligament. The space between the subcutaneous tissue and external oblique muscle, as well as between the external and internal oblique muscles, was dissected appropriately under endoscopic visualization. Arrowheads: external oblique aponeurosis. Asterisks: internal oblique muscle.

Two days later, second-look surgery was performed. The patient’s general condition was stable, the intestinal color was normal, and no evident intra-abdominal contamination was observed. As fascial closure remained difficult, ECS was attempted. On the left side, a 3-cm skin incision was made two fingerbreadths below the costal margin and two fingerbreadths lateral to the rectus abdominis. Under direct vision, the external oblique aponeurosis was incised to expose the internal oblique muscles. The external oblique aponeurosis was further incised craniocaudally within the visible field. In addition, the plane between the external and internal oblique muscles was bluntly dissected under direct visualization. A Lap-Protector (FF0504SD; Hakko medical, Chikuma, Nagano, Japan) was inserted between the external and internal oblique muscles (**Fig. 1A**, **1B**). EZ access (for FF0504 Lap-Protector, Hakko medical) and 5-mm and 12-mm trocars were placed. Carbon dioxide was insufflated to a pressure of 12 mm Hg, resulting in the creation of a space between the external and internal oblique muscles. Under endoscopic guidance, the incision in the external oblique aponeurosis observed on the external oblique aponeurosis was extended using the Sonicision Curved Jaw Cordless Ultrasonic Dissection System (SCDA39; Medtronic, Minneapolis, Minnesota, USA). Subsequently, dissection was performed between the internal and external oblique muscles, and between the external oblique and subcutaneous tissues (**Fig. 1C**). Dissection extended from the inguinal ligament to the costal margin.

**Fig. 2:**
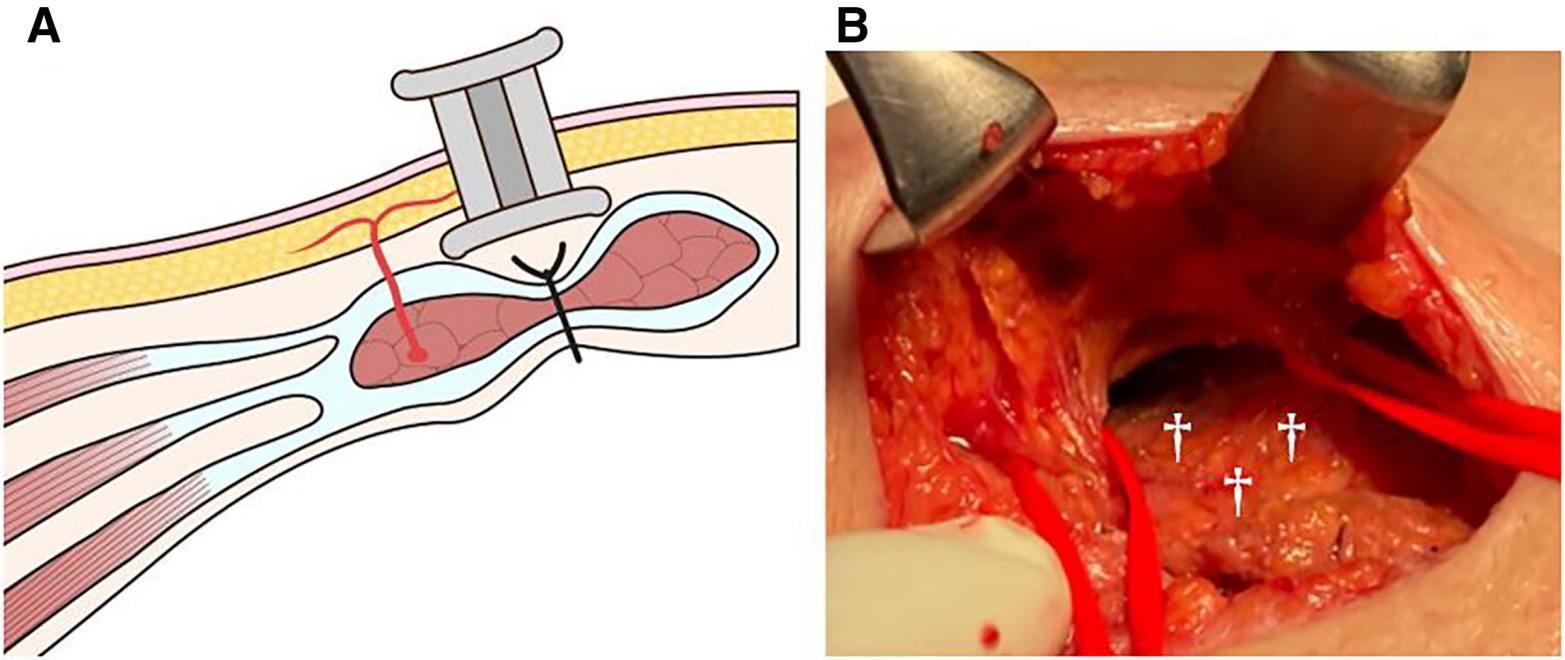
Surgical procedure (right side). (**A**) A stoma site (located above the rectus abdominis muscle) was used. A Lap-Protector was inserted above the rectus abdominis muscle. (**B**) Subcutaneous undermining was performed under direct vision at the level of the external oblique muscle. During this process, preservation of the cutaneous perforator branches (marked with red tape) was confirmed. Daggers: Anterior sheath of the rectus abdominis muscle.

**Fig. 3:**
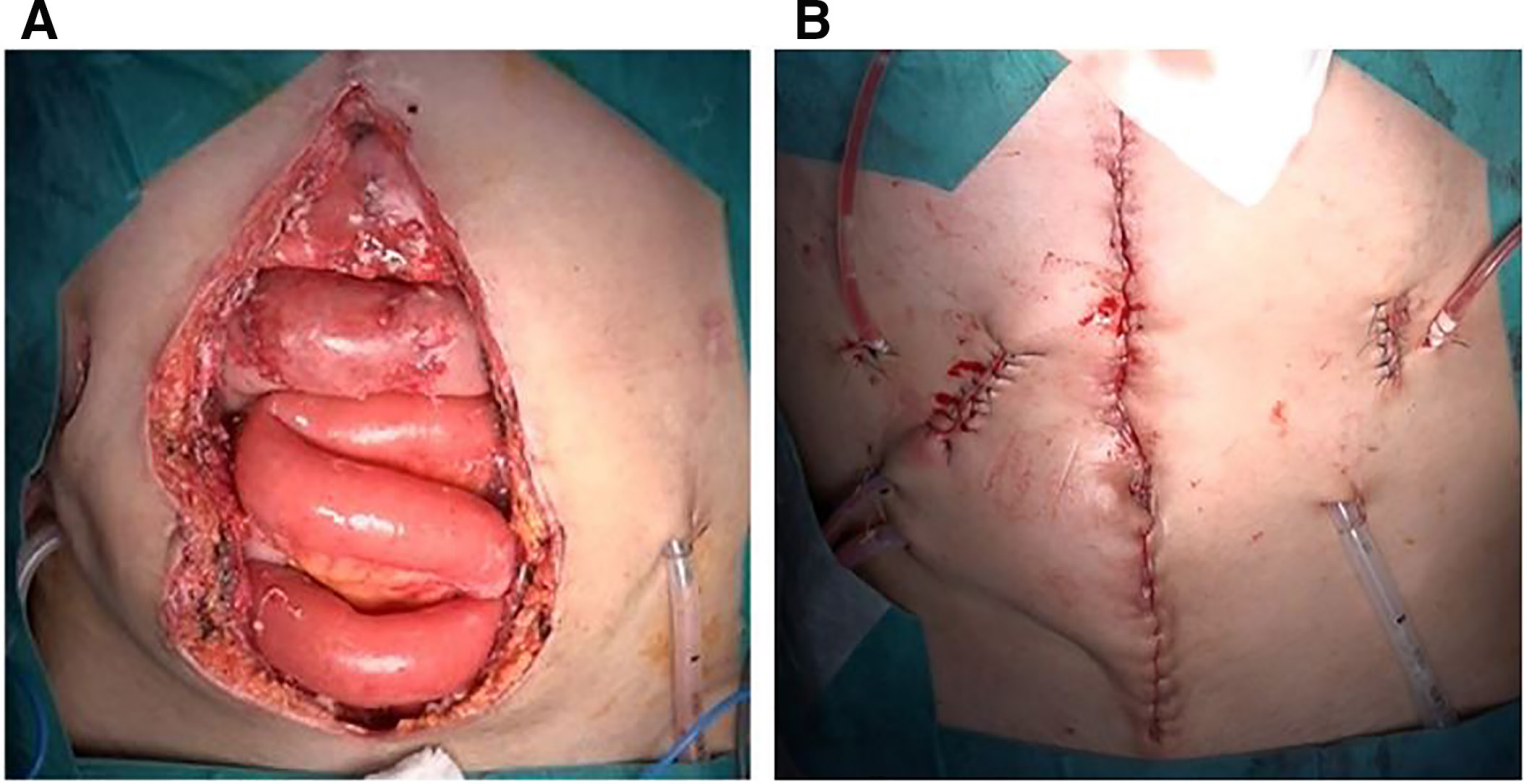
Comparison before and after surgery. (**A**) Preoperatively, there was a gap of approximately 10 cm, making primary fascial closure unfeasible without additional intervention. (**B**) Fascial closure was achieved after ECS. A 7-mm flat drain was placed subcutaneously on both sides. ECS, endoscopic anterior component separation

On the right side, an existing incision (originally intended for stoma creation during the previous surgery; however, no stoma was created, thus leaving only a scar) was used (**Fig. 2A**). Since the wound was located over the rectus abdominis muscle, the subcutaneous tissue was dissected under direct vision to identify the external oblique muscle. Two perforating vessels were preserved in the skin (**Fig. 2B**). A Lap-Protector was placed between the subcutaneous tissue and external oblique muscle (**Fig. 2A**), and the same procedure was performed on the left side. ECS enabled definitive fascial closure (**Fig. 3A**, **3B**). Finally, flat 7-mm drains were placed subcutaneously on both sides. The fascia and peritoneum were sutured together using 0 PDS, and the dermis was closed with continuous suturing using 4-0 STRATAFIX (Ethicon, Somerville, New Jersey, USA). The operative time was 3 hours 1 minute, and the blood loss was 69 mL.

### Postoperative course

**Fig. 4:**
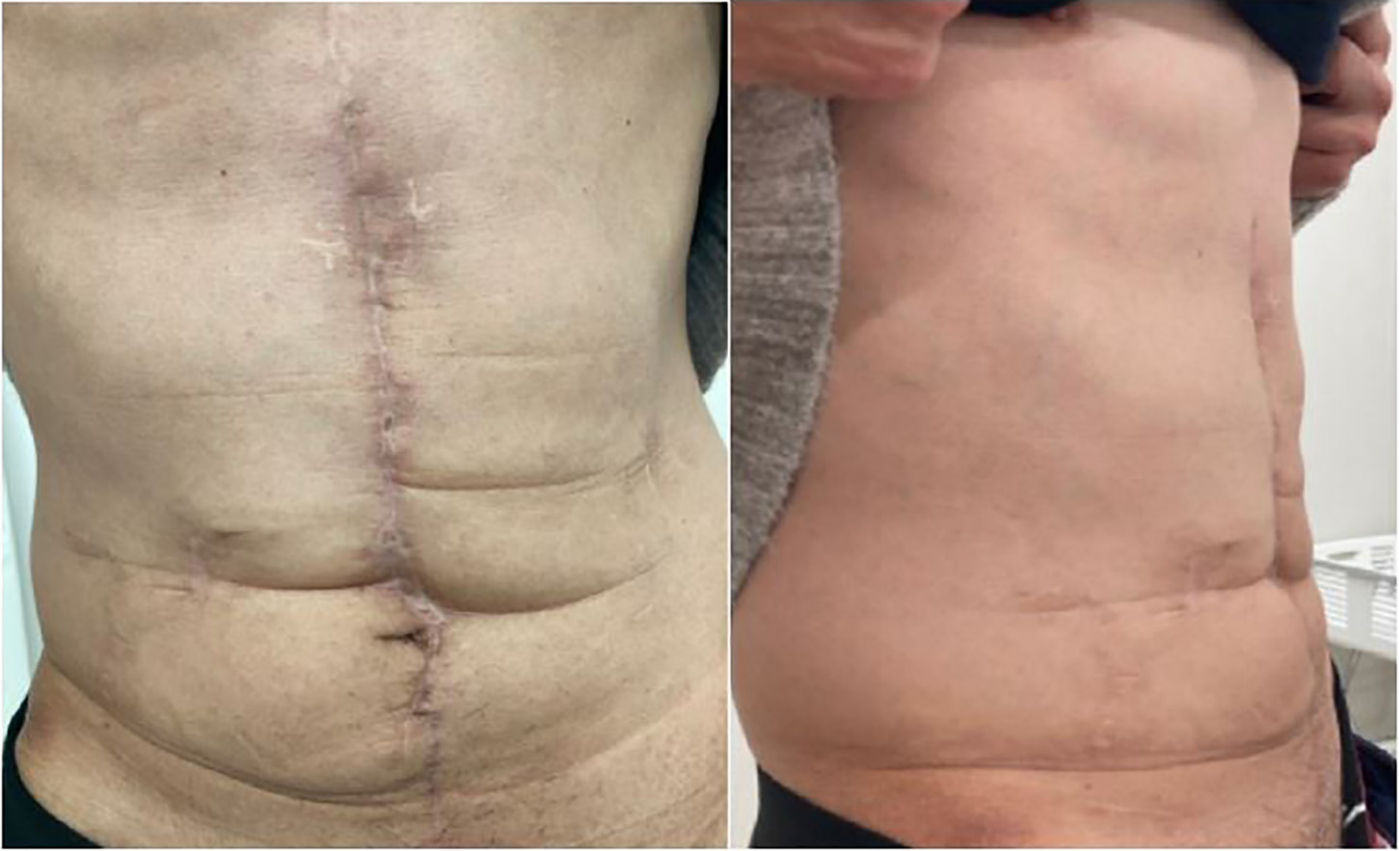
Postoperative course as of 7 months postoperatively, no abdominal wall complications have been observed.

On POD 4, the drain was removed. Although the intra-abdominal abscess required treatment, there were no SSOs. The patient was discharged 40 days after the surgery for colon perforation. Seven months postoperatively, no complications were observed, and the abdominal wall remained stable (**Fig. 4**). At 17 months postoperatively, CT showed no evidence of an incisional hernia, and a well-maintained abdominal wall was successfully preserved.

## DISCUSSION

In recent years, the widespread use of damage control strategies and increased awareness of abdominal compartment syndrome have established OAM as a fundamental approach for managing severe abdominal conditions.^1)^ However, early definitive fascial closure is essential for maintaining fluid and protein homeostasis and for preserving the intraperitoneal environment. It also helps prevent serious complications, such as enterocutaneous fistula and frozen abdomen. However, in clinical practice, abdominal closure is difficult. For patients requiring OAM for more than 24 hours, the probability of achieving definitive closure reportedly decreases by 1.1% with each additional hour.^2)^ Early definitive fascial closure contributes not only to survival and short-term outcomes, but also to QOL, pain reduction, and return to work.^3)^ Ideally, definitive closure should be achieved within 7 days of the initial surgery.^1)^ However, this is not always feasible, and temporary closure is often necessary when a definitive plan is formulated. If early closure is not achievable, a planned ventral hernia may be unavoidable. Nevertheless, the turnover flap technique and component separation methods may enable primary closure even in the early phase of OAM. In this case, a second-look surgery was performed 2 days after surgery due to a colonic perforation, and abdominal closure was successfully achieved on the same day. Although this was a period of intense inflammation and surgical stress, selecting a delayed closure was considered to increase the likelihood of difficulty in abdominal closure. Therefore, early abdominal closure was successfully achieved with the addition of ECS.

OCS was introduced by Ramirez in 1990 as a technique for midline incisional hernia repair.^4)^ In OCS, the subcutaneous tissue is dissected, and the anterior rectus sheath and external oblique aponeurosis are released from the overlying tissue. The external oblique muscle is incised and wide dissection is performed between the external and internal oblique muscles to allow medial advancement of the rectus abdominis and transverse abdominis muscles. This technique does not require mesh, making it suitable for use in infected or contaminated fields. However, OCS involves extensive subcutaneous dissection, which can damage perforating vessels and result in SSOs.^5)^ To address this, ECS was developed by Lowe and Maas.^6,7)^ In ECS, a working space is created between the external and internal oblique muscles, and trocars are placed in this space to incise the external oblique aponeurosis. This minimally invasive approach preserves perforating vessels and reduces the risk of SSOs.^8)^ The width of closure achievable with bilateral ECS is approximately 10–15 cm,^9)^ while that in OCS is approximately 18 cm^4)^; however, some reports suggest that there is no significant difference.^10)^ In the present case, intraoperative findings revealed a fascial defect measuring approximately 10 cm in width, which was not considered small. In OCS, wide dissection between the subcutaneous tissue and rectus abdominis is typically performed in addition to incision of the external oblique aponeurosis. By contrast, dissection between the subcutaneous tissue and external oblique muscle is generally not emphasized in ECS. However, in this case, some degree of dissection between the subcutaneous tissue and the external oblique muscle was performed bilaterally. In comparison to incisional hernia, significant subcutaneous edema was observed after OAM. Subcutaneous dissection may contribute to successful closure after OAM.

In recent years, TAR, a type of posterior component separation, has been reported to be effective in the treatment of large incisional hernias with abdominal wall defects exceeding 10 cm, and is recommended alongside techniques such as ECS.^11)^ No significant differences in SSOs or closure widths have been reported between ECS and TAR.^12,13)^ TAR is generally favored because of its low recurrence rate, as mesh placement is a standard procedure.^14)^

At our institution, more than 10 cases of definitive abdominal closure using OCS following OAM have been previously reported; however, postoperative SSOs remain a significant concern. Therefore, prior to the present case, we attempted abdominal closure using TAR in a single patient. Nevertheless, the expected abdominal wall expansion was insufficient and closure could not be achieved. Ultimately, the abdomen was closed at a later date using a turnover flap. At that time, we opted not to employ OCS because its use would have resulted in the lateral abdominal wall being composed solely of the internal oblique muscle, thereby rendering the abdominal wall fragile and increasing the risk of hernia formation. Conversely, the turnover flap technique involves a longitudinal incision along the outer margin of the anterior rectus sheath, which is then reflected medially using the linea alba as a hinge, thereby creating a flap composed of the anterior sheath to facilitate abdominal closure.^15)^ Although the midline consists only of the anterior rectus sheath, this method is less likely to result in herniation and the musculature of the lateral abdominal wall is preserved. Therefore, in cases where closure with TAR alone was not feasible, we used the turnover flap technique in combination to achieve successful closure. Based on these prior experiences, ECS was selected in the present case. Although no reports have documented the use of TAR for early closure after OAM, the reason for its lack of efficacy remains unclear. One possibility is technical insufficiency: inadequate dissection between the transversus abdominis and transversalis fasciae may have limited lateral release. Another hypothesis is that the TAR may be inherently unsuitable during the early phase of OAM. The transversus abdominis muscle acts in opposition to medial advancement of the abdominal wall, and TAR achieves mobilization by releasing this muscle.^16)^ However, in the early phase of OAM, the abdominal wall is typically edematous owing to fluid resuscitation, inflammation, and injury, leading to third-space fluid accumulation. This edematous, stiff abdominal wall differs significantly from that observed in elective hernia surgery.^17)^ Therefore, subcutaneous edema may be a limiting factor that renders TAR ineffective during early OAM.

Following the present case, we encountered an additional case in which abdominal closure was achieved using ECS following OAM. Although the abdominal wall defect was approximately 5 cm wide, the patient developed hepatic compartment syndrome due to a large hematoma. To avoid compression of the liver, ECS was performed with an additional dissection of the posterior rectus sheath. Tension-free abdominal closure was successfully achieved without any SSOs, and the postoperative liver function remained within normal limits. Even in cases where the abdominal wall defect is not necessarily large, the application of ECS can facilitate tension-free abdominal closure and it may also reduce the risk of abdominal compartment syndrome. Taken together, these findings suggest that abdominal closure using ECS after OAM is an effective strategy for achieving early definitive closure owing to its technical simplicity, flexibility in application, and potential to reduce complications compared with conventional OCS.

## CONCLUSIONS

Early definitive fascial closure is the goal of OAM. In this case, ECS allowed for successful early closure in a minimally invasive manner. Under endoscopic guidance, ECS facilitates precise anatomical dissection while preserving perforating vessels and may help reduce postoperative wound complications.

Further studies are warranted to determine the most appropriate techniques for achieving abdominal closure and minimizing complications after OAM. This case suggests that ECS is an effective and feasible option for early definitive closure.
